# Energetic Profile in Forehand Loop Drive Practice with Well-Trained, Young Table Tennis Players

**DOI:** 10.3390/ijerph17103681

**Published:** 2020-05-23

**Authors:** Yongming Li, Bo Li, Xinxin Wang, Weijie Fu, Boyi Dai, George P. Nassis, Barbara E. Ainsworth

**Affiliations:** 1 School of Physical Education & Sport Training, Shanghai University of Sport, Shanghai 200438, China; libo-1991@foxmail.com (B.L.); 15800867860@163.com (X.W.); georgenassis@gmail.com (G.P.N.); 2 School of Kinesiology, Shanghai University of Sport, Shanghai 200438, China; fuweijie@sus.edu.cn (W.F.); Barbara.Ainsworth@asu.edu (B.E.A.); 3 Division of Kinesiology and Health, University of Wyoming, Laramie, WY 82071, USA; bdai@uwyo.edu

**Keywords:** high repetition practice, energy contribution, energy cost, stroke frequency, oxygen uptake, table tennis

## Abstract

The forehand loop drive is one of the primary attacking techniques in table tennis and is practiced at a large volume during training. The aim of this study was to investigate the energetic profile of the high-repetition forehand loop drive practice in table tennis. Twenty-six well-trained, young table tennis players performed a treadmill graded exercise test to determine their peak oxygen uptake as a measure of overall cardiorespiratory fitness and an incremental table tennis stroke test with 3-min intervals during the forehand loop drive with a ball-throwing robot at a frequency of 35 to 85 strokes∙min^−1^. Pulmonary and blood parameters were measured and analyzed with a portable spirometry system and a blood lactate analyzer. Energy contributions were calculated from aerobic, anaerobic lactic, and anaerobic alactic pathways for each stroke frequency. Energy cost was defined as the amount of energy expended above resting levels for one stroke. Repeated-measures analyses of variance (ANOVA) with the stroke frequency (35,45,55,65,75, or 85 strokes/min^−1^) as a within-subject factor were performed for the dependent variables. A Power regression was performed for the energy cost as a function of the stroke frequency. Findings demonstrated a function of Y = 91.566·x^−0.601^ where Y is the energy cost and x is the stroke frequency, R^2^ = 0.9538. The energy cost decreased at higher stroke frequencies. The energy contributions from aerobic, anaerobic lactic, and anaerobic alactic pathways at each stroke frequency ranged from 79.4%–85.2%, 0.6%–2.1%, and 12.9%–20.0%, respectively. In conclusion, the energy cost of the forehand loop drive decreased at higher stroke frequencies. The high-repetition forehand loop drive practice was aerobic dominant and the anaerobic alactic system played a vital role.

## 1. Introduction

Table tennis is a complex skill that involves a repeated combination of acceleration, deceleration, changing of direction, and balance control in order to produce the optimal strokes [1]. Skilled performers require many years of practice to acquire the techniques needed for success in competitive matches. Table tennis movements can be divided into two parts: footwork and stroke. Footwork involves different movement types (e.g., one step, short steps, and crossover), which are performed at varying frequencies and with great agility [2]. The stroke involves diverse sport-specific techniques (e.g., drive, chop, and block), and is performed at varying frequencies with different types of spin placed on the ball [3]. Among the most important table tennis technique is the forehand loop drive since it is one of the primary attacking techniques and is vital for winning in a rally [4,5,6]. Skilled table tennis players are able to execute the forehand loop drive at varying frequencies throughout the duration of a table tennis game. In order to optimize the table tennis stroke techniques, high-repetition practice (also called multi-ball practice) is used extensively during table tennis training [7,8]. The practice is accompanied with minimal footwork (stationary practice), especially in young athletes, to isolate the stroke skills needed for success in competitive settings [7,8]. High-repetition, stationary practice is reported to account for approximately one-third of the training in the Chinese national team [8].

A table tennis match lasts a maximum of 10 min and is fast paced with intermittent moments of slow-to-very-fast movements [9]. Accordingly, the energetic demands of the game include a combination of aerobic and anaerobic energetic pathways. However, the contribution of each pathway is unclear for specific table tennis skills. Previous studies have explored the energetic profile of table tennis in training and simulated matches [9,10,11,12,13] with findings consistently showing an aerobic-dominant profile. However, the energetic profile in table tennis may be influenced by different physical loads placed on the players such as different footwork, stroke types, and stroke frequencies. A better understanding of the energetic profile in table tennis can be established by investigating the energetic profile for each combination of table tennis footwork and stroke types. Since table tennis players use the forehand loop drive most often to gain a competitive advantage in a match, much of the time in training is spent on developing skills of the forehand loop drive using high repetition practice (e.g., 200 reps). Several studies have explored high-repetition practices from the aspects of dynamic posture control [14], training efficiency [15], and fitness improvement [16]. However, the energetic profile of the high-repetition forehand loop drive has had limited attention [17]. Knowing the detailed energetic profile of the forehand loop drive may help the development of sport-specific training programs to enhance aerobic and anaerobic energetic pathways used in competitive table tennis [10,18].

Energy cost reflects the energy demands of a physical task and is traditionally expressed as the amount of energy spent in an activity above resting levels as needed to transport 1 kg of body mass over 1-m distance (with units of J·m^−1^ or J·kg^−1^·m^−1^). Energy cost has been extensively investigated in different cyclic sports and exercises [19,20]. In general, the energy cost in cyclic exercises where the body movement patterns are repeated (e.g., running, bicycling) is constant when assessed indoors and it increases as the speed of outdoor locomotion increases [19,21,22]. High-repetition stroke training (e.g., 200 reps forehand loop drive practice) in table tennis normally lasts several minutes and, because of the repetitive nature of the movement, it is considered a cyclic exercise. On the other hand, acyclic exercises are those where the body movements change in response to the sport situation (e.g., racket sports). It is inappropriate to apply traditionally defined energy cost equations of cyclic activities to acyclic sports and exercises since the locomotion of the body in acyclic sports and exercises differs from cyclic exercises. Since all racket sports use a racket to stroke and return the ball, there is a need to quantify the energy cost of these unique movements. As such, a modified definition of the energy cost for racket sports is the amount of energy spent above resting levels, which is defined as the energy cost of 1 stroke (with unit of J·stroke^−1^ or J·kg^−1^·stroke^−1^). As a cyclic activity, knowing the energy cost of the forehand loop drive at different stroke frequencies could provide an insight into the energy demands of this frequently used stroke and inform coaches of the best way to structure practice sessions to optimize performance in competitive settings.

The aim of this study was to investigate the energetic profile of the high-repetition forehand drive loop with different stroke frequencies performed with minimal footwork. It was hypothesized that the energy cost would stay in one range as the stroke frequencies increased, and that the energetic profile would be aerobic dominant.

## 2. Materials and Methods

### 2.1. Participants

Twenty-six healthy well-trained young table tennis players from the China Table Tennis College volunteered to participate in this study (Table 1). The players trained for approximately 30 hours per week and competed at the national-level in their age group. Prior to the study, test procedures and all possible risks were described to the subjects and their parents. Informed written consent was provided by their parents in accordance with the declaration of Helsinki. The ethics committee of the Shanghai University of Sport approved all procedures (Approval code: 2015018).

### 2.2. Design and Procedures

The study design was cross-sectional. All participants performed one treadmill graded exercise test and one incremental table tennis stroke test on separate days with at least 24 h spent resting in-between tests. The treadmill graded exercise test was designed to determine the peak oxygen uptake (VO_2peak_) as a reference measure of cardiorespiratory fitness, while the purpose of the incremental table tennis stroke test was to determine the energy cost of the repetitive forehand loop drive at different stroke frequencies.

#### 2.2.1. Treadmill Graded Exercise Test

Participants were well habituated to the laboratory environment and the specific testing protocols prior to any formal tests. They were also instructed to dress and eat a meal as they usually would for matches. A treadmill Bruce Test (Pulsar 3p, h/p/cosmos Sports & Medical GgmbH, Nussdorf, Germany) was utilized to determine VO_2peak_ (Table 1). The test started from a speed of 2.7 km/h and a gradient of 10% with an increase in speed and grade every 3 min [23]. A security belt was used for participants throughout the test. The test was stopped if the participants showed any of the following symptoms: (1) intense exertion (e.g., hyperpnoea, facial flushing, unsteady gait, or participants requested to stop), (2) heart rate within 5% of the age-predicted maximum, (3) heart rate that leveled off over the final stages of the test, or (4) respiratory exchange ratio equaled to or greater than 1.10 [24]. VO_2peak_ was calculated as the average value of VO_2_ during the last 15 s of the test. A heart rate monitor (Polar Accurex Plus, Polar Electro Inc., Kempele, Finland) and a portable spirometry system (K4b^2^, Cosmed, Rome, Italy) were utilized during the test. Standard calibration was strictly performed on each test day prior to the test. A 3-L syringe and a standard gas with a known composition (O_2_: 16.00%, CO_2_: 5.09%) were utilized for the calibration. Prior to the test and 1, 3, 5, 7, and 10 min after the treadmill test, 10 µL of capillary blood was collected from the ear lobe to determine the peak blood lactate concentration (BLC) (Biosen C_line, EKF Diagnostic, Magdeburg, Germany). The altitude, temperature, humidity, and atmosphere pressure for VO_2peak_ and the following step tests were ~5 m, 20 °C, ~60 %, and ~1015 mbar, respectively.

#### 2.2.2. Incremental Table Tennis Stroke Test

During the second visit, participants performed an incremental forehand loop drive test by stroking the table tennis balls rhythmically when served by a robot (V-989H, TaiDe, Shenzhen, China). The balls used in this experiment were standard plastic balls with a diameter of 40 mm. During a 5-min warm up period, participants adjusted their standing position relative to the table and experienced the six different stroke frequencies performed during consecutive 3-minute stages. After a 5-min rest, the test started with a stroke frequency of 35 strokes·min^−1^ (drop point robot setting of level 10, top spin setting of level 8, back spin robot setting of level 2, and loop setting of level 4). The test increased by 10 strokes·min^−1^ every 3 min until reaching 85 strokes·min^−1^ during the sixth stage. There was a 1-min rest period between each stage to allow for collection of blood samples. The robot was adjusted to throw the ball to the corner area of the racket-hand side of the subject. Participants were required to stroke the ball back diagonally to the other side of the table as forcefully as possible with the forehand loop drive. Continuously missing the target area for four times was considered to be technical fatigue, and the test was stopped [25]. All participants finished the designed six stages. A portable spirometry system (K4b^2^, Cosmed, Rome, Italy) was utilized to measure the gas exchange from the warm-up period to 6 min after the end of the last stage. A total of 10 μL capillary blood was taken before the first stage, between each stage, and during the recovery period at 1, 3, 5, 7, and 10 min. The blood was analyzed as in the treadmill graded exercise test with the accumulated blood lactate values used to calculate the energy from the anaerobic lactic pathway. A heart rate monitor (Polar Accurex Plus, Polar Electro Inc., Kempele, Finland) was worn around the chest to assess the heart rate throughout the test. A rating of perceived exertion (RPE) graph was shown to participants immediately after each stage to determine their self-rating of fatigue [26].

#### 2.2.3. Calculation of Energy Contributions and Energy Cost of Exercise

**Figure 1:**
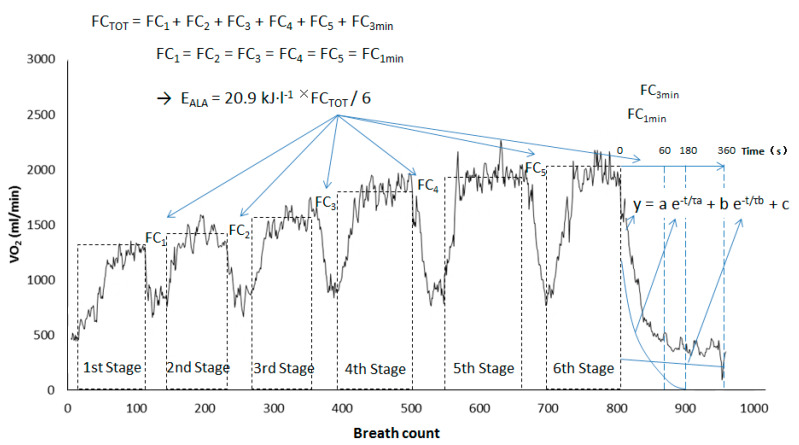
Illustration of the calculation of E_ALA_ in the incremental table tennis stroke test with the raw data (breath by breath) of one subject. Note: FC_TOT_ is the fast component of VO_2_-off kinetics of the whole test (first to sixth stage). FC_n_ is the fast component of VO_2_-off kinetics of the first five stages (FC_1_, FC_2_, FC_3_, FC_4_, FC_5_). FC_3min_ is the fast component of VO_2_-off kinetics in the first 3 min of the recovery. FC_1min_ is the fast component of VO_2_-off kinetics in the first 1 min of the recovery. E_ALA_ is the anaerobic alactic energy contribution of each stage. In y = a e^−t/τa^ + b e^−t/τb^ + c, y—VO_2_ in the recovery after the sixth stage, a and b are the amplitudes of the fast and slow components, respectively, τ_a_ and τ_b_ are the corresponding time constants, and c is the VO_2_ at rest while *t* is time of recovery in seconds.

Energy contributions were calculated as three components for each stage [27,28]. The aerobic energy contribution (E_AER_) was calculated from the accumulated VO_2_ during each stage above resting levels, defined as 3.5 mL·min^−1^·kg^−1^ for females and 4.0 mL·min^−1^·kg^−1^ for males in a standing posture [29]. The anaerobic lactic energy contribution (E_BLC_) was calculated from the accumulated blood lactate during each stage of the forehand loop drive test (post value minus initial value) with the O_2_-lactate equivalent of 3.0 mL·kg^−1^·mM^−1^ [30]. The anaerobic alactic energy contribution (E_ALA_) was calculated from the fast component of the VO_2_ off-kinetics during the rest between each stage, and during the six-min recovery after the sixth stage (Figure 1). The time course of the VO_2_ in the recovery after exercise was interpolated using a bi-exponential equation estimated from a non-linear fitting procedure (Excel 12, Microsoft). The equation is described by:

y = a e^−t/τa^ + b e^−t/τb^ + c (1)

where y is the VO_2_ in the recovery after the sixth stage. a and b are the amplitudes of the fast and slow components, respectively. τ_a_ and τ_b_ are the corresponding time constants, and c is the VO_2_ at rest, while *t* is time of recovery in second [27]. Additionally, it was assumed that the E_ALA_ repayment during the 1-min rest between each stage was similar to that of the first 1 min during the recovery by following the sixth stage, and that the E_ALA_ was similar in each stage [28]. Therefore, the total E_ALA_ of the six stages was calculated from the 3-min fast component of VO_2_ off-kinetics after the sixth stage (corresponding to that of the sixth stage) plus five times of the 1-min fast component of VO_2_ off-kinetics after the sixth stage (corresponding to that of the first five stages). The E_ALA_ of each stage was one-sixth of the total E_ALA_ (Figure 1). Lastly, the fractions of the three energy contributions were calculated from the total VO_2_ (mL) to determine the energy contributions in J or kJ, with an assumed caloric equivalent of 20.9 kJ·L^−1^ (corresponding to a respiratory quotient of 0.96) [20]. The total energy contribution (E_TOT_) was computed as the sum of E_AER_, E_BLC_, and E_ALA_. The energy cost (J·kg^−1^·stroke^−1^) of each stage was calculated by dividing the E_TOT_ of one stage by the corresponding stroke frequency in the same stage and body mass of the player. The equation is: Energy Cost (J·kg^−1^·stroke^−1^) = E_TOT_ (J) /{stroke frequency (strokes·min^−1^) × duration of each stage (min) × body mass (kg)}.

### 2.3. Statistical Analyses

Repeated-measures analyses of variance (ANOVA) with the stroke frequency (35, 45, 55, 65, 75, or 85 strokes·min^−1^) as a within-subject factor were performed for the dependent variables. The ANOVA procedure was not performed for E_ALA_ as this variable was constant for the different stroke frequency conditions. When the sphericity assumption in repeated-measures ANOVAs was violated, the Greenhouse-Geisser correction was performed. When an ANOVA showed a significant main effect, post-hoc paired t-tests were performed between each pair of two stroke frequency conditions using the Benjamini–Hochberg procedure [31] to control the study-wide false discovery rate of 0.05 [31]. A Type-I error rate was set at 0.05 for ANOVAs for statistical significance. In addition, a power regression was performed with the stroke frequency as the predictor and the energy cost as the outcome variables. Statistical analyses were performed using the SPSS Statistics 24 software (IBM Corporation, Armonk, NY, USA).

## 3. Results

The VO_2peak_ of the participants was 2.763 ± 0.651 l·min^−1^ and 47.1 ± 9.6 mL·min^−1^·kg^−1^ (Table 1). The peak BLC after the treadmill test was 10.6 ± 3.4 mM. ANOVAs showed significant main effects for all dependent variables (*p* < 0.05, Table 2). In the post-hoc paired t-tests, the largest p-value for a significant post-hoc paired t-test was 0.034 after the adjustment for the study-wide false discovery rate. A detailed description of the perceived and energetic characteristics at each stage is provided in Table 2. The percentage of VO_2peak_ from the first stage to the last stage of the table tennis forehand drive loop test increased from 46.5 ± 14.0% to 60.0 ± 18.0%. The fractions of E_AER_, E_BLC_, and E_ALA_ between the first and sixth stage ranged from 79.4 ± 5.8% to 85.2 ± 5.1%, from 0.58 ± 0.74% to 2.13 ± 2.92%, and from 12.9 ± 3.7% to 20.0 ± 5.9%, respectively.

**Figure 2:**
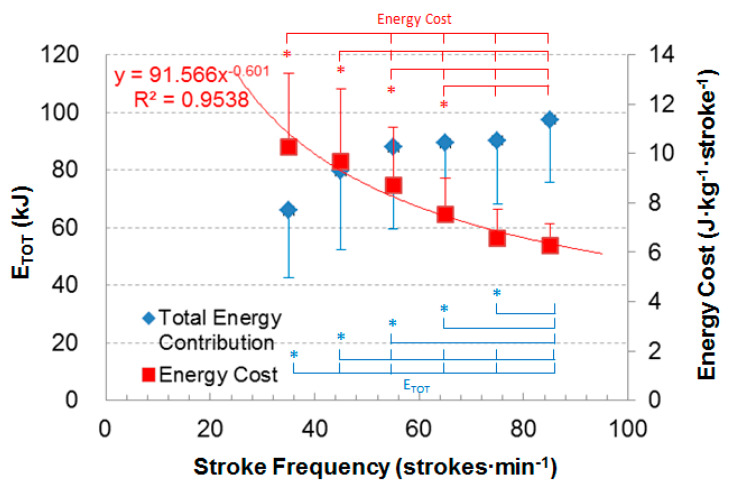
Total energy contribution and energy cost of the forehand loop drive at different stroke frequencies. Note: E_TOT_—total energy contribution. Y = 91.566·x^−0.601^—the function of power regression for the energy cost related to the stroke frequency, where Y is energy cost, and x is the stroke frequency. *—significantly different from other stages (*p* ≤ 0.05).

As Figure 2 shows, the total energy contribution (E_TOT_) in kJ increased significantly from 35 to 85 strokes·min^−1^, except for the stage from 55 to 75 strokes·min^−1^. The energy cost in J·kg^−1^·stroke^−1^ decreased significantly from 35 to 85 strokes·min^−1^, except from 35 to 45 strokes·min^−1^ and from 75 to 85 strokes·min^−1^. The power regression of the energy cost of the forehand loop drive practice as related the stroke frequency in young table tennis players was Y = 91.566·x^−0.601^ (R^2^ = 0.9538), where Y is energy cost in J·kg^−1^·stroke^−1^, and x is stroke frequency of the forehand loop drive.

## 4. Discussion

Our results partially rejected the hypothesis given that the energy cost declined as the stroke frequency increased. However, the high-repetition forehand loop drive practice was aerobic dominant and this confirms our second hypothesis. The increasing stroke frequency in the incremental table tennis forehand loop drive test resulted in an elevation in physiological measures of effort (e.g., HR, VO_2_, BLC) and an increase in the perceptual measure of fatigue (RPE) during each stage. The energy cost of the forehand loop drive decreased with each incremental stage. Since the intensity of the test was manipulated by increasing the stroke frequency in each 3-min stage, the sum of strokes made during the test increased from approximately 105 to 255 strokes from the first to the sixth stage.

The decrease of the energy expended per stroke might be attributed to a decreased force exertion and a shorter range of stroke motion since the speed of the forehand drive increased. The balls were thrown by a robot at a constant frequency that required a higher demand of quickness and physiological responses for players at higher stroke frequencies. Considering the frequency of the forehand loop drive increased from 35 to 85 strokes·min^−1^, the time window of each stroke for players decreased from ~2.0 to 0.7 s. This required a rapid response in returning the ball. Although the players were instructed to stroke the ball back with a high quality return, they might have modified the quality of their stroke (i.e., stroke force and range of motion) in order to compensate for the frequency of the ball delivery [32,33]. This could have reduced the energy expended during successive stages of the incremental test. If this hypothesis is true, this possible decrease of stroke quality (i.e., increased errors) in high repetition performance at a higher stroke frequency should be understood by coaches when they design high repetition forehand loop drive training with a high stroke frequency. Accordingly, an individual’s highest stroke frequency without modification of stroke quality should be identified for each player before designing high repetition practices.

Fatigue might be a potential reason for the decrease of the energy expended per stroke at the higher stroke frequencies. However, none of the participants were eliminated from completing the test by missing the target area in four continuous balls indicating that technical fatigue was not present in the study [25]. Nevertheless, fatigue during the later stages of the incremental forehand loop drive test may have played a role in a possible decrease in the velocity of the ball return. Our initial study design called for measuring the energetic profile of the repeated forehand loop drive for 3 min at each stroke frequency on different days or with a 10-min rest period between successive stroke frequencies to avoid fatigue during the latter stages of an incremental test. However, the participant’s busy academic and training schedules made the original study design too time-consuming and logistically unrealistic. Therefore, measurements for the six stroke frequencies (35–85 strokes·min^−1^) were combined into one incremental test with six stages. Since the participants were required to stroke the ball as forcefully and accurately as possible during the test, physical and mental fatigue might have occurred during successive stages of the test [1,34]. However, acknowledging that the anaerobic threshold was not measured, the BLC measures during the stroke test appeared to be below the metrics for the anaerobic threshold [35,36]. Hence, the possible fatigue effect in the incremental stroke test may not have been due to physical fatigue, but due to mental fatigue from the intense concentration needed to return the ball delivered at high frequencies. It appears that the tasks in the incremental stroke test were sustainable for the well-trained players in the study, but it might lead to higher levels of mental and physical fatigue in beginners or untrained players, especially the mental fatigue.

A unique aspect of this study is calculating the energy contributions from aerobic and alactic systems during the high repetition forehand loop drive in the table tennis test. Table tennis is considered an aerobic dominant sport with the anaerobic alactic system playing a significant role during the rallies [10,12,37]. The findings from this study are consistent with the literature [9,10,11,12,13,37], wherein the percentage of energy contributions of E_AER_, E_BLC_, and E_ALA_ range from 79.4–85.2%, 0.6–2.1%, and 12.9–20.0%, respectively, in this study (Table 2). However, in simulated matches, Zagatto et al. found different percentages for E_AER_, E_BLC_, and E_ALA_ of 96.5%, 1.0%, and 2.5%, respectively [10]. The differences between the studies were likely induced by the duration of the stages (5.7 min in Zagatto vs. 3 min in this study) and the mode of the workload (intermittent in Zagatto vs. continuous in this study). It has been demonstrated previously that the relative energy contributions of physical exertion are correlated with the duration of the workload with higher %E_AER_ in longer duration stages than shorter duration stages [38,39]. To evaluate the efficacy of a prediction formula to predict the %E_AER_ of the table tennis forehand loop drive provided by Li et al. [39] (y = 23.355 × e^x^ + 41.02, where y is %E_AER_, and x is duration of a high intensity workload) was applied to the data obtained in this and Zagatto’s study. The %E_AER_ were both lower than values in the studies (current study, 66.6%, and Zagatto, 81.5%). It is possible that the underestimation of %E_AER_ computed with the Li et al. formula may be due to the high-intensity, continuous cyclic sports (e.g., cycling, running) used to develop the formula [39]. Although the intensity during the rallies in table tennis was high, the duration of the rallies was short (~3–5 s) [9,40], and the rally to the rest ratio was low (~1:2) [9]. This is a fact that has reduced the overall intensity of table tennis. Additionally, the findings from this study support the importance of the anaerobic alactic energy system during the rallies, and the limited contribution of the anaerobic lactic energy system with the %E_ALA_ and %E_BLC_ values in Table 2. Even though the players in this study were well-trained, national-level table tennis players, the aerobic-dominance of the 3-min forehand loop drive can be applied to players of differing skill levels since the 3-min stages was much longer than the threshold duration of the aerobic-anaerobic dominance (i.e., about 75 s) [38,39].

The uniqueness of this study is the introduction of a new definition of the energy cost for table tennis, provided by a function of energy cost for the table tennis high-repetition forehand loop drive test (Y = 91.566·x^−0.601^, R^2^ = 0.9538). This equation can be used to estimate the energy cost of the forehand loop drive practice in young table tennis players if the measured energy cost is not possible. Such information could influence training methods based on different metabolic responses between the upper-body and lower-body exercise modes [41].

The study also had some limitations which should be noted. The primary finding of the energetic profile in this study was based on the calculation of the energy contributions using the method integrated by Beneke et al. [27]. This method calculates the anaerobic alactic fraction of energy based on the fast component of VO_2_-off kinetics [27]. Normally, at least 6 min is needed for calculating the fast component of VO_2_-off kinetics. However, it would be time-consuming for the subjects to take such long breaks between each stage or to perform the six stages on separate days, and it would present an unrealistic setting of a table tennis performance. Therefore, it was difficult to calculate the anaerobic alactic fraction of energy since the breaks between each stage was 1 min in this study. Accordingly, we assumed that the E_ALA_ repayment during the 1-min rest between each stage of the incremental table tennis test was roughly similar to that of the first 1 min during the recovery after the sixth stage, and that the E_ALA_ was similar in each stage, as recommended by Davis et al. [28]. Although this assumption might overestimate the E_ALA_ for the first five stages, this overestimate was considered to be minimal, given that the intensity of all the six stages was moderate (HR < 153 bpm and RPE < 6, Table 2). In addition, the decrease of the energy cost in the forehand loop drive at increased stroke frequencies was postulated to be associated with a decreased force exertion and shorter range of motion with increasing stroke speeds. However, this postulation needs to be verified with kinetic and kinematic data in the future. Moreover, the high-repetition forehand loop drive with different stroke frequencies was performed incrementally instead of randomly. Although this design might induce a fatigue effect in later stages, this effect was considered to be limited given the relatively low intensity of the forehand loop drive. Lastly, we utilized the %VO_2peak_ as one of the indicators of intensity in the forehand loop drive. It is recognized that the muscle mass and techniques used in the forehand loop drive are very different from the treadmill test. However, future studies should consider using an arm ergometer [42] or a maximal incremental stroke test with a ball-throwing robot to determine the VO_2peak_ [43]. Nevertheless, the VO_2peak_ determined from a treadmill graded exercise test is an excellent indicator of one’s overall cardiorespiratory fitness, which can be compared with values found in other studies.

Future studies should expand the knowledge of the energy cost of the forehand loop drive to other table tennis techniques, such as performing stroke skills with different footwork patterns and with a combination of other table tennis techniques. The energy cost of the forehand loop drive as explored in this study could be utilized to determine the individual threshold of different stroke frequencies with or without substantial technique deterioration and to determine the individual threshold of one’s stroke frequency as an indicator for performance in the longitude training diagnostics. Lastly, this study should be repeated in table tennis players with skills lower than those included in this study to determine the generalizability of the results.

## 5. Conclusions

In conclusion, the energy cost of the forehand loop drive decreased at higher stroke frequencies. This may have practical implications for table tennis coaches and athletes to avoid modifications in this technique during high repetition practice. In addition, the high repetition forehand loop drive task was demonstrated to be aerobically dominant with the anaerobic alactic energy system playing a vital role, and the anaerobic lactic system having limited importance.

## Figures and Tables

**Table 1:** Participants’ characteristics.

	Units	Female	Male	All-Pooled
Sample size		12	14	26
Age	years	17.1 ± 2.0	17.2 ± 2.6	17.2 ± 2.3
Height	cm	163.7 ± 3.9	172.9 ± 7.8 *	168.6 ± 7.8
Body Mass	kg	56.5 ± 6.0	64.2 ± 10.4 *	60.6 ± 9.4
VO_2peak_	L·min^−1^	2.404 ± 0.306	3.071 ± 0.718 *	2.763 ± 0.651
	mL·min^−1^·kg^−1^	44.4 ± 5.5	49.4 ± 11.7 *	47.1 ± 9.6
Training Experience	years	8.0 ± 2.3	9.2 ± 2.2	8.7 ± 2.2

VO_2peak_—peak oxygen uptake determined in a treadmill graded exercise test, *—significantly different from females, *p* < 0.05.

**Table 2:** Perceived, physiological, and energetic characteristics of forehand loop drive practice (mean ± SD).

Stage	1	2	3	4	5	6
Stroke frequency (strokes·min^−1^)	35	45	55	65	75	85
RPE	0.92 ± 0.88 ^a^	1.71 ± 1.3 ^b^	2.67 ± 1.81 ^c^	3.50 ± 2.25 ^d^	4.33 ± 2.41 ^e^	5.42 ± 2.7 ^f^
HR (bpm)	115.4 ^a^± 13.8	127.1 ± 12.8 ^b^	137.6 ± 15.9 ^c^	141.8 ± 17.1 ^c^	144.7 ± 17 ^d^	152.9 ± 18 ^e^
VO_2_ (mL·min^−1^)	1291.8 ± 444.4 ^a^	1484.3 ± 442.6 ^b^	1605.5 ± 441.4 ^c^	1631.6 ± 420.3 ^c^	1629.2 ± 366.7 ^c^	1664.9 ± 387.3 ^c^
VO_2_ (mL·min^−1^·kg^−1^)	21.2±5.6 ^a^	24.5 ± 6.2 ^b^	26.4 ± 5.3 ^c^	26.8 ± 4.8 ^c^	26.9 ± 4.3 ^c^	27.4 ± 4.1 ^c^
%VO_2peak_ (%)	46.5 ± 14 ^a^	53.6 ± 16.3 ^b^	58.6 ± 18.2 ^c^	59.8 ± 18.8 ^c^	60.0 ± 18 ^c^	60.7 ± 15.7 ^c^
BLC (mM)^#^	1.30 ± 0.38 ^a^	1.41 ± 0.43 ^a^	1.67 ± 0.6 ^b^	1.78 ± 0.71 ^b^	1.88 ± 0.84 ^b^	2.46 ± 0.98 ^c^
E_AER_ (kJ)	52.8 ± 20.5 ^a^	66.6 ± 24.2 ^b^	74.2 ^b^ ± 24.8	76.2 ± 21.1 ^b^	77.2 ± 20.5 ^b^	82.9 ± 18.9 ^c^
E_BLC_ (kJ)	0.55 ± 1.04 ^a^	0.56 ± 0.85 ^a^	1.13 ± 1.24 ^b^	0.60 ± 1.12 ^a^	0.56 ± 0.92 ^a^	2.09 ± 3.29 ^b^
E_ALA_ (kJ)	12.5 ± 4.1	12.5 ± 4.1	12.5 ± 4.1	12.5 ± 4.1	12.5 ± 4.1	12.5 ± 4.1
E_AER_ (%)	79.4 ± 5.8 ^a^	83.0 ± 4.3 ^b^	84.2 ± 3.13 ^c^	85.2 ± 3.5 ^d^	85.2 ± 5.1 ^b^	85.0 ± 4.3 ^c^
E_BLC_ (%)	0.63 ± 1.03 ^a^	0.58 ± 0.74 ^a^	1.20 ± 1.07 ^b^	0.66 ± 1.14 ^a^	0.58 ± 0.9 ^a^	2.13 ± 2.92 ^c^
E_ALA_ (%)	20.0 ± 5.9 ^d^	16.4 ± 4.5 ^c^	14.6 ± 3.2 ^b^	14.2 ± 3.5 ^b^	14.3 ± 5.3 ^b^	12.9 ± 3.7 ^a^

Note: a, b, c, d, e and f are significance symbols, and the values with these symbols are significantly larger or smaller according to the relationship of a < b < c < d < e < f, *p* ≤ 0.034. RPE—rating of perceived exertion. HR—heart rate. VO_2_—oxygen uptake. %VO_2peak_—percentage of VO_2peak_. BLC—blood lactate concentration after each stage. E_AER_—aerobic energy contribution. E_BLC—_anaerobic lactic contribution. E_ALA_—anaerobic alactic contribution. *n* = 26, except for RPE (*n* = 24) and HR (*n* = 20). #—the resting value before the first stage is 1.24 ± 0.32 mM.

## References

[1] Girard O., Millet G.P. (2009). Neuromuscular fatigue in racquet sports. Phys. Med. Rehabil. Clin. N Am..

[2] Malagoli Lanzoni I., Lobietti R., Merni F. Footwork techniques used in table tennis: A qualitative analysis. Proceedings of the 10th ITTF Sports Science Congress.

[3] Munivrana G., Furjan-Mandi G., Kondri M. (2015). Determining the structure and evaluating the role of technical-tactical elements in basic table tennis playing systems. Int. J. Sport Sci. Coach..

[4] Xiao D., Wang Z., Tang J., Su P. (2013). Kinematics character of lower limbs when the table tennis players using attack and loop drive technique of positive hand. J. Shenyang Sport Univ..

[5] Zhang X., Zhang L., Xiao D. (2009). Kinematical character of table tennis player’s upper limbs in forehand fast attack and loop drive technique. China Sport Technol..

[6] Tepper G., Leandro O. (2003). ITTF Level 1 Coaching Manual.

[7] Xiong Z. (2009). Design of a teaching plan for multiple Ping-Pong ball training. J. Physic. Educ..

[8] Gu L., Liu Y. (2006). Investigation on multi-ball training methods in table tennis. China Sport Coach..

[9] Zagatto A.M., Morel E.A., Gobatto C.A. (2010). Physiological responses and characteristics of table tennis matches determined in official tournaments. J. Strength Cond. Res..

[10] Zagatto A.M., De Mello Leite J.V., Papoti M., Beneke R. (2016). Energetics of table tennis and table tennis specific exercise testing. Int. J. Sports Physiol. Perform..

[11] Shieh S.-C., Chou J.-P., Kao Y.-H. (2010). Energy expenditure and cardiorespiratory responses during training and simulated table tennis match. Int. J. Table Tennis Sci..

[12] Sperlich B., Koehler K., Holmberg H.-C., Zinner C., Mester J. (2011). Table tennis: Cardiorespiratory and metabolic analysis of match and exercise in elite junior national players. Int. J. Sports Physiol. Perform..

[13] Milioni F., Leite J.V.M., Beneke R., de Poli R.A.B., Papoti M., Zagatto A.M. (2018). Table tennis playing styles require specific energy systems demands. PLoS ONE.

[14] Gu Y., Yu C., Shao S., Baker J.S. (2019). Effects of table tennis multi-ball training on dynamic posture control. PeerJ.

[15] Cao Z., Xiao Y., Lu M., Ren X., Zhang P. (2020). The impact of eye-closed and weighted multi-ball training on the improvement of the stroke effect of adolescent table tennis players. J. Sports Sci. Med..

[16] Katsikadelis M., Pilianidis T., Mantzouranis N., Berberidou F., Fatouros I. (2017). The influence of 10 weeks high-intensity interval Multiball training on aerobic fitness in adolescent table tennis players. Biol. Exerc..

[17] Katsikadelis M., Pilianidis T., Mantzouranis N., Fatouros I., Agelousis N. (2014). Heart rate variability of young table tennis players with the use of the multiball training. Biol. Exerc..

[18] Zagatto A.M., Kondric M., Knechtle B., Nikolaidis P.T., Sperlich B. (2018). Energetic demand and physical conditioning of table tennis players. A study review. J. Sports Sci..

[19] Di Prampero P.E. (1986). The energy cost of human locomotion on land and in water. Int. J. Sports Med..

[20] Di Prampero P.E., Salvadego D., Fusi S., Grassi B. (2009). A simple method for assessing the energy cost of running during incremental tests. J. Appl. Physiol..

[21] Zamparo P., Bonifazi M., Bagchi D., Sreejayan N., Sen C.K. (2013). Bioenergetics of Cyclic Sport Activities in Water. Nutrition and Enhanced Sports Performance: Muscle Building, Endurance, and Strength.

[22] Zamparo P., Capelli C., Pogliaghi S., Bagchi D., Sreejayan N., Sen C.K. (2013). Bioenergetics of Cyclic Sport Activities on Land. Nutrition and Enhanced Sports Performance: Muscle Building, Endurance, and Strength.

[23] Haff G., Dumke C. (2012). Laboratory Manual for Exercise Physiology.

[24] Armstrong N., Welsman J.R. (2001). Peak oxygen uptake in relation to growth and maturation in 11- to 17-year-old humans. Eur. J. Appl. Physiol..

[25] Zagatto A.M., Papoti M., Gobatto C.A. (2008). Validity of critical frequency test for measuring table tennis aerobic endurance through specific protocol. J. Sports Sci. Med..

[26] Armstrong N. (2007). Paediatric Exercise Physiology.

[27] Beneke R., Pollmann C., Bleif I., Leithauser R.M., Hutler M. (2002). How anaerobic is the wingate anaerobic test for humans?. Eur. J. Appl. Physiol..

[28] Davis P., Leithäuser R., Beneke R. (2013). The energetics of semi-contact 3 x 2 min amateur boxing. Int. J. Sports Physiol. Perform..

[29] Ciba-Geigy (1985). Wissenschaftliche Tabellen Geigy (Scientific Tables Geigy).

[30] Di Prampero P.E. (1981). Energetics of muscular exercise. Rev. Physiol. Biochem. Pharm..

[31] Benjamini Y., Hochberg Y. (1995). Controlling the false discovery rate—A practical and powerful approach to multiple testing. J. R Stat. Soc..

[32] Iino Y., Kojima T. (2016). Effect of the racket mass and the rate of strokes on kinematics and kinetics in the table tennis topspin backhand. J. Sports Sci..

[33] Xiao D., Su B., Tang J. (2008). GRF of loop drive technique of table tennis players. J. Tianjin Univ. Sport.

[34] Le Mansec Y., Pageaux B., Nordez A., Dorel S., Jubeau M. (2018). Mental fatigue alters the speed and the accuracy of the ball in table tennis. J. Sports Sci..

[35] Borg G. (1970). Perceived exertion as an indicator of somatic stress. Scand J Rehabil Med.

[36] Heck H., Mader A., Hess G., Muecke S., Mueller R., Hollmann W. (1985). Justification of the 4-mmol/l lactate threshold. Int. J. Sports Med..

[37] Kondrič M., Zagatto A.M., Sekulić D. (2013). The physiological demands of table tennis: A review. J. Sports Sci. Med..

[38] Gastin P.B. (2001). Energy system interaction and relative contribution during maximal exercise. Sports Med..

[39] Li Y., Niessen M., Chen X., Hartmann U. (2015). Overestimate of relative aerobic contribution with maximal accumulated oxygen deficit: A review. J. Sports Med. Phys. Fit..

[40] Yuza N., Sasaoka K., Nishioka N., Matsui Y., Yamanaka N., Ogimura I., Takashima N., Miyashita M. (1992). Game analysis of table tennis in top Japanese players of different playing styles. Int. J. Table Tennis Sci..

[41] Reis V.M., Neves E.B., Garrido N., Sousa A., Carneiro A.L., Baldari C., Barbosa T. (2019). Oxygen uptake on-kinetics during low-intensity resistance exercise: Effect of exercise mode and load. Int. J. Environ. Res. Public Health.

[42] Zagatto A.M., Papoti M., Reis I.G.M.D., Beck W.R., Gobatto C.A. (2014). Analysis of cardiopulmonary and metabolic variables measured during laboratory and sport-specific incremental tests for table tennis performance prediction. Sci. Sports.

[43] Zagatto A., Miranda M., Gobatto C. (2011). Critical power concept adapted for the specific table tennis test: Comparisons between exhaustion criteria, mathematical modeling, and correlation with gas exchange parameters. Int. J. Sports Med..

